# Supplementary material for: Spontaneous Primary Succession and Vascular Plant Recovery in the Iberian Gypsum Quarries: Insights for Ecological Restoration in an EU Priority Habitat
Source: Plants (Basel). 2023 Mar 3;12(5):1162. doi: 10.3390/plants12051162 (PMC10006988; doi:10.3390/plants12051162)
Supplement: Supplementary file 1 [file plants-12-01162-s001.zip › plants-2219688-supplementary figures.pdf]

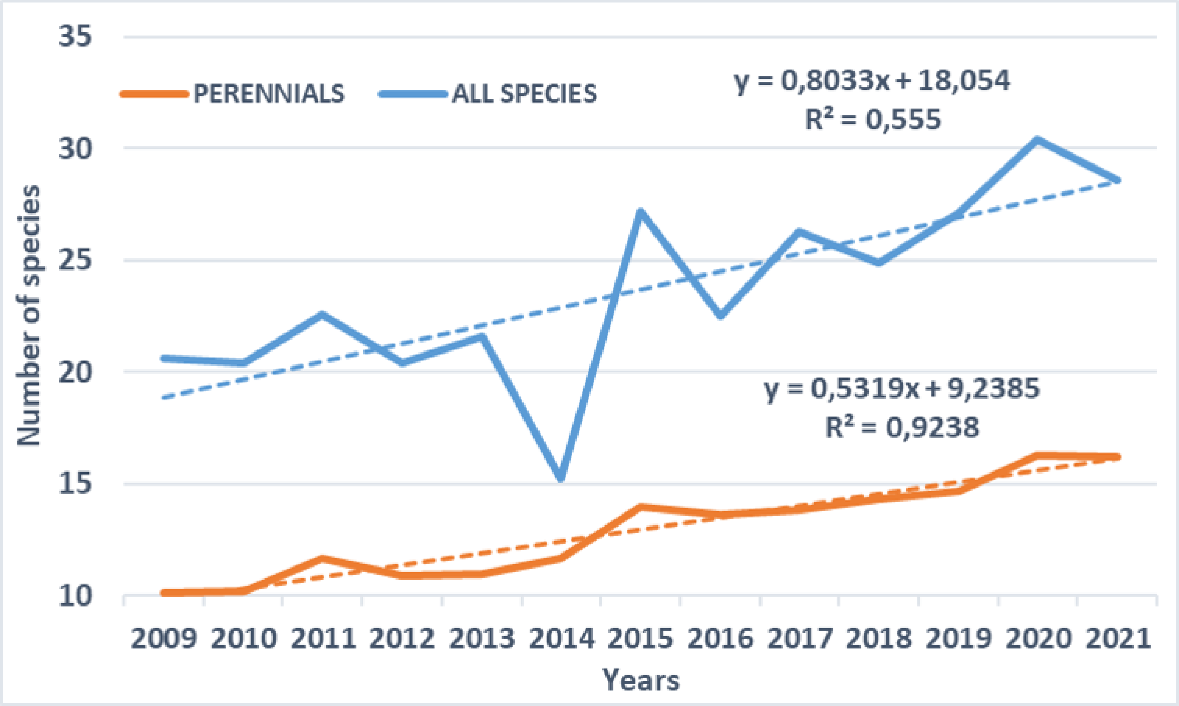

Figure S1: Variation in species richness throughout the chronosequence.

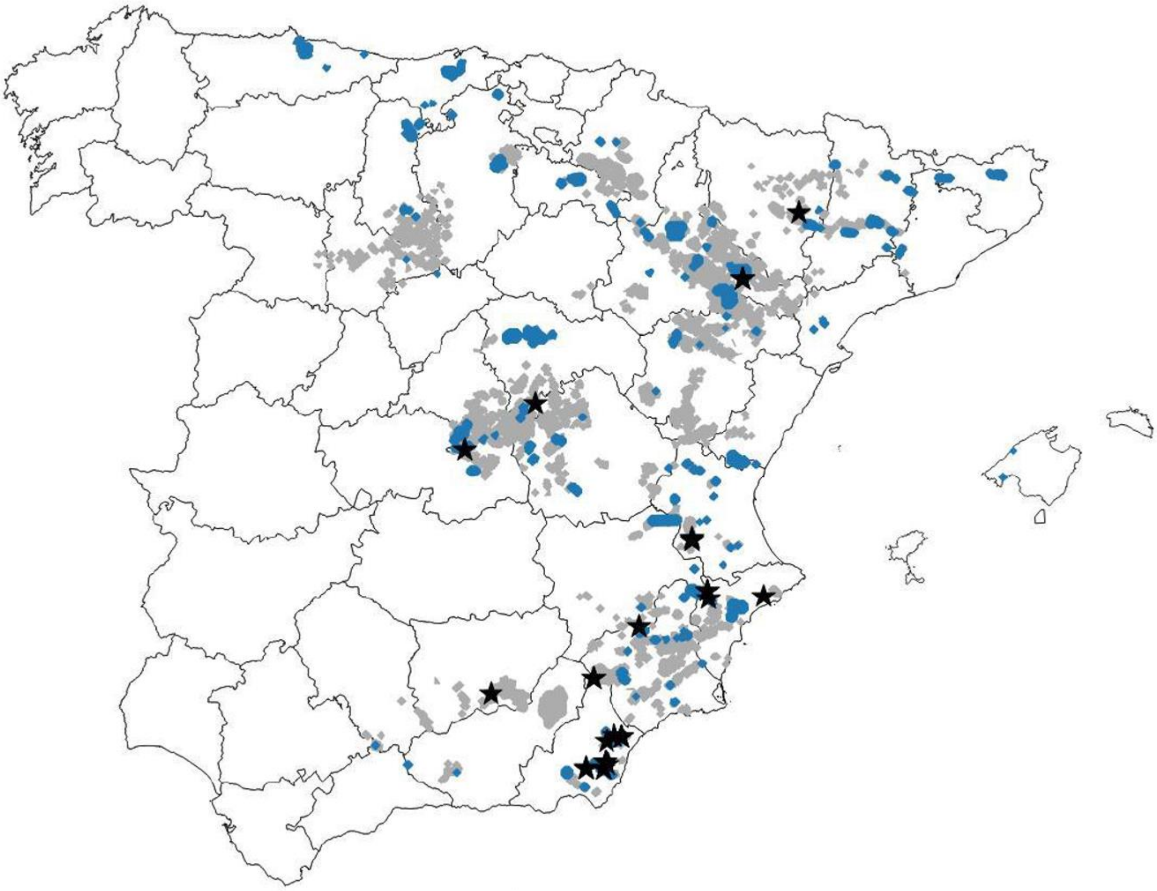

Figure S2: Location of the sampled gypsum quarries in which spontaneous succession processes were recognized (black stars). Grey, Iberian gypsum outcrops with gypsicolous vegetation [48], and blue Spanish gypsum mining concessions (https://www.igme.es/).
